# Transcriptional regulation of long-term memory in the marine snail *Aplysia*

**DOI:** 10.1186/1756-6606-1-3

**Published:** 2008-06-17

**Authors:** Yong-Seok Lee, Craig H Bailey, Eric R Kandel, Bong-Kiun Kaang

**Affiliations:** 1 National Creative Research Initiative Center for Memory, Department of Biological Sciences, College of Natural Sciences, Seoul National University, San 56-1, Silim-dong, Gwanak-gu, Seoul 151-747, Korea; 2 Howard Hughes Medical Institute, Columbia University College of Physicians and Surgeons, New York, NY 10032, USA; 3 Center for Neurobiology and Behavior, Columbia University College of Physicians and Surgeons, New York, NY 10032, USA

## Abstract

Whereas the induction of short-term memory involves only covalent modifications of constitutively expressed preexisting proteins, the formation of long-term memory requires gene expression, new RNA, and new protein synthesis. On the cellular level, transcriptional regulation is thought to be the starting point for a series of molecular steps necessary for both the initiation and maintenance of long-term synaptic facilitation (LTF). The core molecular features of transcriptional regulation involved in the long-term process are evolutionally conserved in *Aplysia, Drosophila*, and mouse, and indicate that gene regulation by the cyclic AMP response element binding protein (CREB) acting in conjunction with different combinations of transcriptional factors is critical for the expression of many forms of long-term memory. In the marine snail *Aplysia*, the molecular mechanisms that underlie the storage of long-term memory have been extensively studied in the monosynaptic connections between identified sensory neuron and motor neurons of the gill-withdrawal reflex. One tail shock or one pulse of serotonin (5-HT), a modulatory transmitter released by tail shocks, produces a transient facilitation mediated by the cAMP-dependent protein kinase leading to covalent modifications in the sensory neurons that results in an enhancement of transmitter release and a strengthening of synaptic connections lasting minutes. By contrast, repeated pulses of 5-hydroxytryptamine (5-HT) induce a transcription- and translation-dependent long-term facilitation (LTF) lasting more than 24 h and trigger the activation of a family of transcription factors in the presynaptic sensory neurons including ApCREB1, ApCREB2 and ApC/EBP. In addition, we have recently identified novel transcription factors that modulate the expression of ApC/EBP and also are critically involved in LTF. In this review, we examine the roles of these transcription factors during consolidation of LTF induced by different stimulation paradigms.

## Introduction

Memory can be divided into declarative and non-declarative processes. Declarative or explicit memory is the conscious recall of knowledge about facts and events and is particularly well developed in the vertebrate brain. Non-declarative or implicit memory is the non-conscious recall of skilled behavior and other tasks and includes simple associative forms such as classical conditioning and non-associative forms such as sensitization [1,2]. During sensitization an animal learns about the properties of a single noxious stimulus enabling the formation of a learned fear response. The cellular and molecular mechanisms that underlie the storage of implicit memory have been most extensively analyzed in the gill- and siphon-withdrawal reflex of marine mollusk *Aplysia*. This organism offers several unique advantages for the study of learning and memory, such as a relatively simple and tractable central nervous system, large identified neurons, and well-characterized neural circuits related to specific behaviors that can be modified by learning [3]. In *Aplysia*, sensitization of the gill- and siphon-withdrawal reflex is induced by a strong stimulus to its tail [4]. Repetitive stimuli produce long-term sensitization that lasts days to several weeks whereas a single stimulus induces short-term sensitization lasting only minutes to a few hours [5,6]. These two forms of memory can be reconstituted in dissociated sensory-motor neuron cultures by the modulatory neurotransmitter serotonin (5-HT) [7]. A single pulse of 5-HT induces short-term facilitation (STF), whereas five applications of 5-HT induce long-term facilitation (LTF) [8].

In contrast to the short-term synaptic changes, which involve covalent modifications of preexisting molecules leading to an alteration of preexisting synaptic connections, long-term synaptic changes require the synthesis of new macromolecules including mRNAs and proteins [8-11]. In addition, long-term changes are accompanied by structural modifications including the growth of new synaptic connections between the sensory neurons and their target motor neurons [12,13]. These features of long-term, learning-related synaptic plasticity, are highly conserved throughout evolution of the nervous system. A variety of experimental systems, ranging in complexity from *Aplysia *to rodents, have been used to examine the molecular mechanism underlying long-term synaptic changes [1,2,10,11]. In this review, we focus on the role of nuclear transcription factors in the presynaptic sensory neurons of *Aplysia *during LTF.

## A model system for examining the molecular biology of long-term memory – the *Aplysia* sensory to motor neuron synapse

In sensory-motor neuron cultures, STF and LTF can be induced by applying 5-HT. One pulse of 5-HT activates PKA and PKC, probably via the activation of different types of G proteins. Activated PKA phosphorylates a potassium channel (S channel), resulting in the elevated influx of calcium leading to an increase in membrane excitability and spike broadening. PKC facilitates the mobilization of synaptic vesicles to the presynaptic active zone. Together, these kinases enhance transmitter release by modifying preexisting molecules [14-17].

LTF induced by repeated pulses of 5-HT requires the synthesis of both new proteins and RNAs. Inhibitors of protein or RNA synthesis selectively block LTF, but not STF when applied within a critical time window that encompasses the training protocol [8]. Analogous to STF, cAMP-dependent protein phosphorylation is also involved in LTF and cAMP analogs induce LTF [18]. Bacskai and colleagues first demonstrated that in response to repeated pulses of 5-HT the catalytic subunit of PKA translocates into the nucleus of the presynaptic sensory neuron to activate CREB-1 [19]. Repeated pulses of 5-HT also induce phosphorylation of MAPK and the activated MAPK also translocates into the nucleus of the sensory neuron where it removes the repressive influence of ApCREB-2 [20].

Dash et al. provided the first evidence of the involvement of cAMP-inducible genes expressed during LTF in *Aplysia*. Microinjection of CRE (cAMP-responsive element) oligonucleotides into the nucleus of sensory neurons selectively blocked 5-HT-induced LTF without affecting short-term changes [21]. These data first suggested that LTF requires the activation of cAMP-inducible genes, and that CRE oligonucleotides prevent interactions between CRE-binding protein (CREB)-related transcription factors and these genes. Using a newly developed gene transfer technique, Kaang and colleagues next showed directly that four or more pulses of 5-HT stimulate CRE-mediated gene expression. Moreover, transcription induced by 5-HT requires the phosphorylation of CREB on Ser^119 ^by PKA [22]. Indeed Bartsch et al. went on to show that injection of phosphorylated CREB into the sensory neuron can by itself initiate the long-term process [23]. Collectively these results suggested that a signaling axis composed of cAMP-PKA-CREB participates in the molecular cascade leading to the expression of LTF. Subsequent studies confirmed the involvement of a number of related downstream molecules in the induction of LTF, including CAMAP, ApC/EBP, and ApAF.

## ApCREBs – Central modulators of LTF

In *Aplysia*, ApCREB1a and ApCREB2 have been characterized as an activator and repressor of LTF, respectively. ApCREB2 is a homolog of mammalian CREB2/ATF4 which is also identified as a transcriptional repressor [24,25]. Inhibition of ApCREB2 by injecting antiserum or double-strand RNA (dsRNA) into sensory neurons allowed a single pulse of 5-HT to produce translation-dependent LTF and the growth of new synapses [25,26]. Conversely, injection of an anti-CREB1 antibody into sensory neurons selectively blocked LTF. Moreover, introduction of the phosphorylated transcriptional activator, ApCREB1a, was sufficient to induce LTF. [23]. PKA-mediated activation of ApCREB1a stimulated the downstream transcription factor, ApC/EBP, via recruiting CBP and subsequently facilitating histone acetylation [27]. For activation of downstream gene expression by ApCREB1a, the inhibitory constraint of ApCREB2 must be relieved. This de-repression is mediated by the phosphorylation of ApCREB2 by nuclear translocated MAPK [20,28]. Together these results suggest that LTF requires not only the activation of memory-enhancer genes but also the inactivation of memory-suppressor genes. Moreover, CREBs appear to play a critical role in maintaining the dynamic balance between these positive and negative factors.

CREB, a basic leucine zipper transcription factor, is also reported to be involved in long-term plasticity in the nervous systems of other organisms, including fly and mouse. In transgenic *Drosophila*, expression of dCREB2b led to the blockage of long-term memory, whereas dCREB2a facilitated memory [29,30]. Similarly, CREB-deficient mice displayed impaired long-term potentiation (LTP) and long-term memory [31]. The threshold for late phase LTP was lowered in the hippocampus from mice expressing the constitutively active form of CREB [32].

## ApC/EBP – A key downstream gene for LTF

In view of the critical roles of CREB in memory consolidation, characterization of its downstream effectors has been a major focus of research in both invertebrate and vertebrate learning models [33-35]. In *Aplysia *the CCAAT enhancer-binding protein (ApC/EBP), an immediate early gene during the consolidation phase of LTF, was found by Alberini et al. to be a downstream target of ApCREB1 [36]. The C/EBP family of transcription factors contains a basic leucine-zipper domain. Specifically, expression of ApC/EBP is rapidly induced in response to 5-HT treatment and this occurs to an immediate early gene in a translation-independent manner [36]. Inhibition of ApC/EBP by injection of ERE oligonucleotides, ApC/EBP antiserum or dsRNA into the sensory neurons in sensory-to-motor neuron cultures during a critical time window blocked 5-HT-induced LTF [36,37]. Moreover, a single pulse of 5-HT which normally induces only STF, produces LTF when ApC/EBP is overexpressed in the sensory neuron [37]. These findings support the idea that ApC/EBP is both necessary and sufficient to consolidate short-term memory into long-term memory. However, since overexpression of ApC/EBP alone does not induce LTF, an additional component must be required for converting STF to LTF. Lee and colleagues used the RNA interference technique designed to block the function of ApC/EBP and similarly found that this blocked LTF in *Aplysia *[26,37]. Moreover, in addition to these studies in *Aplysia*, C/EBPβ and -δ are induced in the rodent hippocampus after inhibitory avoidance learning, suggesting that C/EBPs are highly conserved molecular components of the CREB-dependent signal pathway involved in memory consolidation [38].

ApC/EBP is also known to be induced by the neural activity. The depolarization-induced ApC/EBP induction appears to be mediated by transient induction of the nucleolar protein, ApLLP, which was recently characterized as a novel transcription factor induced by neural activity in *Aplysia *sensory neuron [39]. Kim *et al. *also showed that a single pulse of 5-HT can produce LTF when the synapse is pretreated with high potassium solution. LTF induced by this protocol was completely blocked by the injection of anti-ApLLP or anti-ApC/EBP antibody [39].

One important feature of immediate early genes such as ApC/EBP, is that their expression is tightly regulated within a specific and narrow time window. ApC/EBP mRNA displays a peak expression at 2 h after induction that rapidly returns to the basal levels [36]. Recent studies have found that the ApC/EBP 3' UTR contains putative AU-rich element (ARE) sequences which are cis-acting regulatory elements commonly found in labile mRNAs [40]. Different sets of ARE binding proteins may interact with ApC/EBP mRNA to regulate its stability and/or translatability [41-43]. Yim et al. found that an ARE binding protein, ApELAV binds to and stabilizes ApC/EBP mRNA, suggesting that post-transcriptional regulation of ApC/EBP may also play an important role during LTF [40].

## ApAF – A binding partner for both ApCREB2 and ApC/EBP

Both ApC/EBP and ApCREBs are transcription factors containing the basic leucine zipper (bZIP) domain in the C-terminus. This domain is involved in both DNA binding and multimerization [23,25,44]. Using the bZIP domain of ApC/EBP as bait, Bartsch and colleagues screened a cDNA library to identify additional transcription factors acting downstream of ApCREB1. In this fashion, they cloned a novel transcription factor ApAF, whose activity is regulated by PKA [45]. In contrast to ApC/EBP, which is induced in response to 5-HT, ApAF is a constitutively expressed gene. Interestingly, an *in vitro *binding assay revealed that ApAF interacts with both ApCREB2 and ApC/EBP, but not ApCREB1. Inhibition of ApAF by injection of a specific antibody blocked LTF induced by repeated pulses of 5-HT, suggesting that ApAF is necessary for LTF. Previously, it had been found that injection of phosphorylated ApCREB1a by itself or anti-ApCREB2 antibody combined with a single pulse of 5-HT induced LTF that phenocopied 5 × 5-HT-induced LTF [23,25]. ApAF is involved in both forms of LTF: an anti-ApAF antibody blocked LTF induced by phosphorylated CREB1a as well as that by ApCREB2 antibody injection paired with one pulse of 5-HT. Moreover, overexpression of ApAF enhanced both LTF induced by 5 × 5-HT and ApCREB2 antibody with 1 × 5-HT [45]. Thus, ApAF may be a potential memory enhancer gene downstream of ApCREB1 and ApCREB2.

To determine whether ApC/EBP is the critical partner of ApAF, Lee and colleagues investigated the effects of silencing the ApAF gene on LTF induced by ApC/EBP overexpression paired with one pulse of 5-HT [46]. ApAF inhibition by dsRNA completely blocked LTF induced by both ApC/EBP overexpression and 5 × 5-HT. In combination with a single pulse of 5-HT, the ApAF-ApC/EBP heterodimer produced LTF, even in the absence of CRE- and CREB-mediated gene expression. These results provide direct evidence that the ApAF-ApC/EBP heterodimer is a key downstream effecter of ApCREB. Furthermore, ApAF enhances ERE-mediated gene expression by cooperating with ApC/EBP and phosphorylation at Ser-266 of ApAF by PKA is required for activation of the ApAF-ApC/EBP heterodimer during 5-HT-induced LTF [46]. These data explain why ApC/EBP overexpression in the absence of 5-HT could not convert STF to LTF. The single pulse of 5-HT in ApC/EBP-induced LTF possibly functions in triggering the phosphorylation of ApAF by activated PKA.

Lee et al. also examined the role of Ser-266 phosphorylation of ApAF in the relief of ApCREB2 repression [46]. Overexpression of a dominant negative mutant of ApAF which cannot be phosphorylated at Ser-266 completely blocked both LTF induced by 5 × 5-HT and that by the ApC/EBP overexpression combined with 1 × 5-HT. Moreover, this mutant restored ApCREB1-mediated gene expression and 5-HT-induced LTF repressed by ApCREB2 as efficiently as its wild type counterpart, suggesting that Ser-266 phosphorylation of ApAF is not required to relieve ApCREB2 repression [46]. However, the precise signaling pathway that regulates the interactions between ApAF and ApCREB2 remains to be characterized. Taken together, these studies of ApAF indicate that transcriptional regulation of memory consolidation is quite diverse and can recruit both direct and indirect interactions between transcription factors.

## CAMAP – A retrograde signal from the membrane to the CRE promoter

Neurons display a distinct highly differentiated form which consists of three basic compartments: a cell body or soma which contains the nucleus harboring genomic information and two types of processes axons and dendrites. Dendrites are input elements of the neuron. Together with the cell body they receive synaptic contacts from other neurons. Axons are the output elements of the neuron. The branches of each axon form numerous synaptic connections with other neurons. It is well known that long-term synaptic plasticity requires the synthesis of both new RNAs and proteins. This raises three fundamental questions: 1) Does synaptic plasticity always occur in a cell-wide manner? 2) If not, how does the nucleus identify the correct synapses for delivery of gene products to achieve synapse-specific plasticity? 3) Is the nucleus the only site responsible for transcription and translation?

To address the question of synapse specificity, Martin and her colleagues developed a new culture system in *Aplysia *consisting of a bifurcated *Aplysia *sensory neuron which makes synapses with two spatially separated motor neurons in culture [47]. They found that 5-HT can induce branch-specific LTF that was dependent on CREB activation in the nucleus of presynaptic sensory neuron. Branch-specific LTF was also accompanied by synapse-specific growth of new sensory neuron synapses. These studies highlight the importance of a retrograde signal propagating from the stimulated synaptic site to the nucleus. The CREB-downstream molecules produced in the cell body can be captured by other synapses which have been tagged or marked. This synaptic mark is PKA-dependent; however, rapamycin-dependent local protein synthesis is required for LTF to persist for more than 72 h [47,48]. Further molecular studies have dissected the characteristics of the retrograde signal and the synaptic mark.

An early step in the growth of new synaptic connections is the internalization of an NCAM immunoglobulin-related cell adhesion molecule – apCAM. In recent studies Lee et al. addressed the question: How does this internalization of apCAM relate to the activation of transcription? Lee found that CAMAP is an interacting partner of apCAM in the sensory neuron. CAMAP serves as a transcriptional co-activator that is also a crucial retrograde signaling molecule involved in the initiation phase of LTF [49]. ApCAM is down-regulated upon application of 5-HT [50]. Serotonin leads to clathrin-mediated endocytosis of the transmembrane isoform of apCAM (TM-ApCAM) from the surface membrane of sensory neurons and this internalization depends on phosphorylation of its cytoplasmic tail by MAPK [51,52]. When TM-apCAM was overexpressed in sensory neurons, five pulses of 5-HT failed to produce synaptic facilitation or enhancement of synaptic growth, suggesting that down-regulation of apCAM is required for both LTF and the presynaptic structural changes induced by 5-HT [53]. Whereas MAPK is known to be involved in apCAM downregulation, the subcellular mechanisms responsible for apCAM internalization remain to be characterized. CAMAP (apCAM-Associated Protein) was cloned by yeast two-hybrid screening using the cytoplasmic tail of TM-ApCAM as bait [49]. CAMAP is colocalized with apCAM at the surface of the plasma membrane in the basal state and is translocated to the nucleus of sensory neuron after treatment with 5-HT. Nuclear translocation and dissociation from apCAM are modulated by PKA-mediated phosphorylation of CAMAP. A phosphorylation-mimicking mutant of CAMAP can dissociate from apCAM and translocate to the nucleus in the absence of 5-HT stimulation [49]. How does CAMAP translocate to the nucleus? Importins, which transport cargo molecules from the cytosol to nucleus, are critically involved in synaptic plasticity in both *Aplysia *and rodent brain [54]. This raises the possibility that importin may transport CAMAP into the nucleus of the sensory neuron.

In view of nuclear translocation of CAMAP from the plasma membrane, Lee et al. suggested that CAMAP acts as a retrograde signaling molecule in the induction of LTF. To act as a retrograde signal, a molecule must meet several criteria, specifically, 1) it should translocate into the nucleus from the synaptic site in response to stimuli that induce synaptic plasticity, such as LTF and LTP, 2) it should play a specific function in the nucleus, and 3) blockage of its translocation and function should inhibit the long-term synaptic change. CAMAP appears to fulfill all of the above requirements. First, CAMAP translocates to the nucleus following both cell-wide and synapse-specific applications of 5-HT. After 5-HT treatment, the mobility of CAMAP is significantly increased at the distal neurites of sensory neurons. Second, CAMAP binds to ApCREB1 in the nucleus, where it acts as a transcriptional co-activator that can induce ApC/EBP expression. Interestingly, the N-terminal region, but not full-length CAMAP displayed transcriptional activity. The C-terminus of CAMAP appears to act as an autoinhibitory domain. This inhibitory clamp is relieved by PKA phosphorylation of CAMAP. Details of the molecular mechanisms that underlie the co-activator function of CAMAP and the PKA phosphorylation-dependent restoration of transcriptional activity of the N-terminus of CAMAP are currently not known. Finally, blockage of CAMAP expression by dsRNA completely suppresses 5-HT-induced ApC/EBP upregulation and LTF. Moreover, CAMAP dsRNA blocks synapse-specific LTF induced by local application of 5-HT at the synapse. Taken together, these results indicate that CAMAP is a critical retrograde messenger in the initiation of LTF.

The precise role of CAMAP in the internalization of apCAM remains unclear. We have recently found that overexpression of mutant CAMAP that cannot be phosphorylated by PKA impaired the 5-HT-induced internalization of apCAM [49]. This finding suggests that CAMAP tethers apCAM to the plasma membrane in the basal state, and that the phosphorylation and nuclear translocation of CAMAP are necessary for the subsequent down-regulation of apCAM.

## Chromatin alteration and epigenetic changes in gene expression with memory storage

Although epigenetic mechanisms were widely known to be involved in the formation and long-term storage of cellular information in response to transient environmental signals, the discovery of their putative relavance in adult brain function is relatively recent [27,55]. The epigenetic marking of chromatin, such as histone modification, chromatin remodeling and the activity of retrotransposons, may have long-term consequences in the transcriptional regulation of specific loci involved for long-term synaptic changes [56].

The contribution of histone tail acetylation, a modification that favors transcription and is associated with active loci, to LTF formation was first revealed by the study of Guan et al. in *Aplysia *neurons [27]. This study found that both facilitatory and inhibitory stimuli bidirectionally alter the acetylation stage and structure of promoters driven by the expression of genes involved in the maintenance of LTF, such as ApC/EBP. It also demonstrated that enhancing histone acetylation with histone deacetylase (HDAC) inhibitors facilitates the induction of LTF (Guan et al. 2002). HDAC inhibitors have recently been shown to have similar effects during L-LTP in the Schaffer collateral pathway of mammals and to enhance memory formation in hippocampus-dependent tasks [57-61]. HDAC inhibitor, sodium butyrate, has been shown to induce the growth of new dendrites and synapses, which might be an underlying mechanism of its memory enhancing effects [61]. Moreover, mice with reduced histone acetyltransferase activity, such as different mouse models for Rubinstein-Taybi mental retardation syndrome, have deficits in both long-lasting forms of memory and LTP [57,58,62,63]. These results indicate that critical chromatin changes occur during the formation of long-term memory and that these changes are required for the stable maintenance of these memories.

### Perspectives

**Figure 1:**
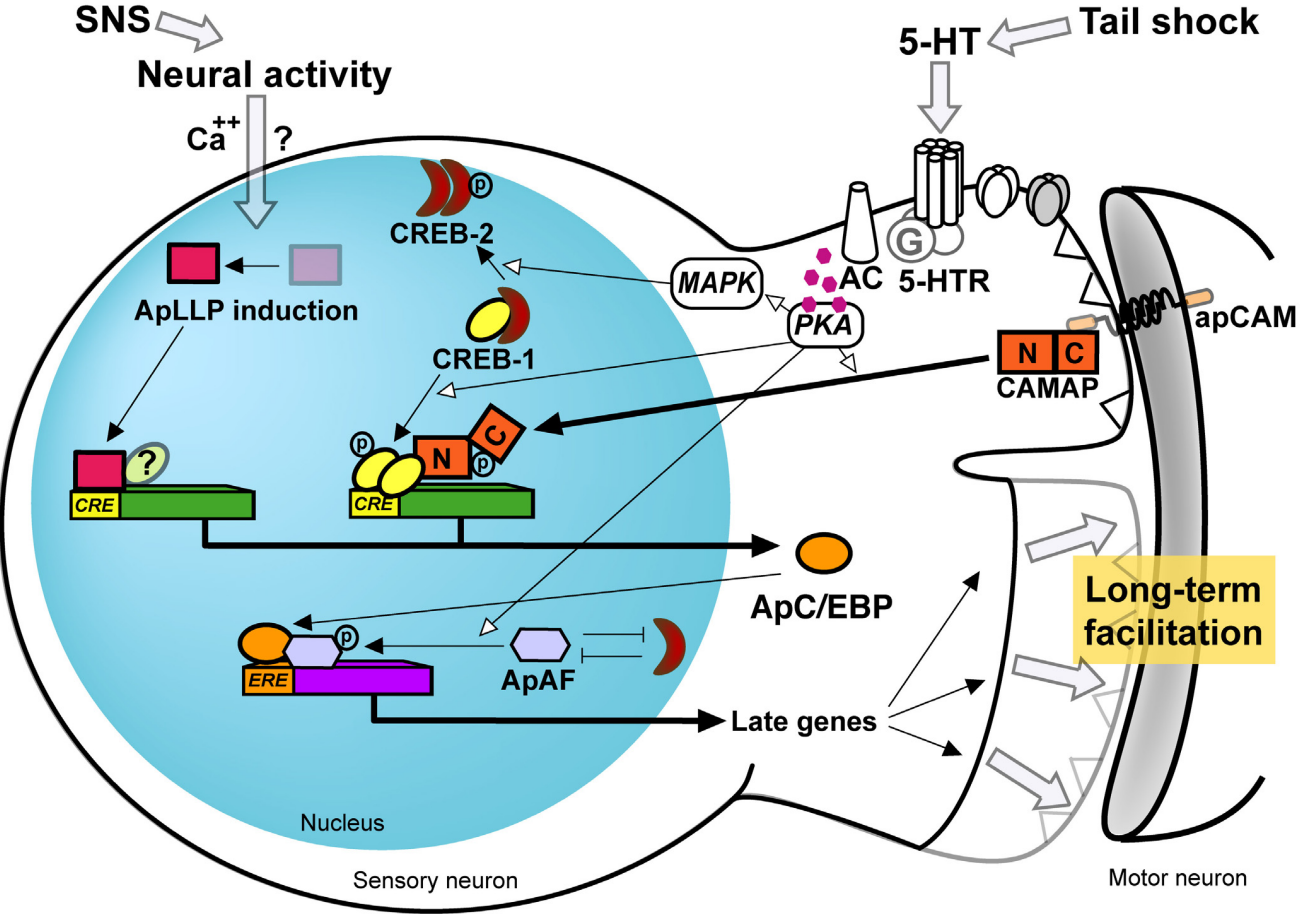
**Schematic model of signaling pathways underlying long-term facilitation in *Aplysia *sensory neuron**. The repeated treatments with neurotransmitter 5-HT activate a G-protein coupled receptor that stimulates adenylyl cyclase, which in turn activates PKA. MAPK are also activated and translocates into the nucleus. At the synaptic site, PKA stimulates the nuclear translocation of the retrograde signal molecule CAMAP via phosphorylating its Ser^148^. This phosphorylation results in both the dissociation from TM-apCAM and the restoration of its transcriptional activity from autoinhibition. In the nucleus, MAPK phosphorylates CREB2 which represses CREB1 and ApAF in the absence of 5-HT. Once freed from CREB2 and stimulated by PKA, CREB1 forms a homodimer to activate the downstream target gene, ApC/EBP. Translocated CAMAP acts as a co-activator of CREB1. ApC/EBP interacts with ApAF that is activated by PKA to form a core downstream effector of CREB1. ApC/EBP-ApAF heterodimer induces the late genes which are critical for the consolidation and maintenance of LTF. Robust neural activity induces and activates the transcription factor, ApLLP in the nucleus in a calcium-dependent manner. ApLLP induces ApC/EBP expression and lowers the threshold for LTF induction. Elucidating the downstream molecule of ApC/EBP remains to be challenged. SNS, strong noxious stimulus.

During the last decade molecular studies have increased our understanding of the signaling and regulatory mechanisms that underlie LTF (Figure 1). Several research groups have generated a variety of cellular models of long-term memory by investigating 5-HT-induced LTF and other forms of long-term synaptic changes induced by different stimulation paradigms in *Aplysia*.

Recently, operant conditioning was demonstrated in the gill-withdrawal and feeding behavior in *Aplysia *and an electrophysiological study revealed that operant and classical conditioning of feeding behavior differentially modify the intrinsic excitability of an identified neuron [64-66]. PolyADP-ribose-polymerase 1 (PARP1) facilitates the transcription of long-term memory related genes by decondensing chromatin structure in neurons that mediate operant conditioning [67]. However, the transcription factors involved in this behavioral modification are yet to be identified.

Interestingly, ApC/EBP is the common downstream molecule of the novel transcription factors, ApLLP and CAMAP. ApC/EBP, which pairs with ApAF, could activate the transcription of effector genes critically involved in the consolidation and maintenance of long-term memory [36]. Genes encoding structural proteins, such as clathrin light chain and the chaperon BiP were identified as late effector genes [68,69]. The elongation factor 1 alpha was also suggested to be essential for maintaining newly formed synapses [70]. However, the number of late genes identified thus far represents only a beginning. Since the DNA-binding motifs of ApC/EBP and ApAF homodimer or heterodimer have been analyzed, completion of the ongoing *Aplysia *genome sequencing project should facilitate the identification of other novel late effector molecules [45].

Recently, advances have also been made in clarifying the molecular mechanisms that contribute to learning-related synaptic plasticity in the mammalian brain, particularly those that underlie the induction and expression of LTP and LTD [71,72]. When it comes to transcriptional regulation, however, our current understanding is far from complete. *Aplysia *was the first organism in which cAMP was shown to play a critical role in learning-related synaptic plasticity [28,73]. Since signaling cascades that underlie the expression of long-term memory are surprisingly well conserved throughout the species, insights from the molecular studies of *Aplysia *should provide an important foundation for future studies into the transcriptional regulation of memory formation in the more complex mammalian brain.

## Authors' contributions

All authors participated in developing the ideas, the writing, discussion and integration of the information. All authors read and approved the final manuscript.
